# Managing Metabolic Dysfunction-Associated Steatotic Liver Disease (MASLD) in the Digital Era: Overcoming Barriers to Lifestyle Change

**DOI:** 10.7759/cureus.84803

**Published:** 2025-05-25

**Authors:** Stanislav Kravchuk, Mykola Bychkov, Marko Kozyk, Olena Strubchevska, Aleksandra Kozyk

**Affiliations:** 1 Gastroenterology, Danylo Halytsky Lviv National Medical University, Lviv, UKR; 2 Internal Medicine, Corewell Health William Beaumont University Hospital, Royal Oak, USA; 3 Internal Medicine, Jagiellonian University Medical College, Krakow, POL; 4 Business, Willamette University, Portland, USA

**Keywords:** behavioral change, dietary intervention, lifestyle modification, metabolic dysfunction–associated steatotic liver disease (masld), metabolic health, non-alcoholic fatty liver disease (nafld), obesity, physical activity, type 2 diabetes, weight loss

## Abstract

Obesity presents a significant global health challenge due to its association with a range of systemic disorders. One such condition is metabolic dysfunction-associated steatotic liver disease (MASLD), formerly known as non-alcoholic fatty liver disease (NAFLD), which has become the most common chronic liver disease worldwide.

The prevalence of MASLD is closely linked to obesity, type 2 diabetes, and sedentary lifestyles, posing substantial risks for liver-related morbidity and extrahepatic complications such as cardiovascular and renal diseases. Despite recent advancements in pharmacological treatments, lifestyle modification remains the cornerstone of MASLD management.

This review summarizes current evidence on lifestyle interventions, focusing on weight loss, dietary improvements, and increased physical activity. It also explores barriers to implementation, including socioeconomic factors, limited patient awareness, and stigma, while highlighting innovative strategies such as group-based programs, web-based interventions, and mobile technologies. As MASLD continues to rise globally, prioritizing lifestyle-based and scalable approaches will be critical to reducing its burden and improving patient outcomes.

## Introduction and background

According to the latest estimates, more than a billion people worldwide are living with obesity, which poses a substantial health challenge because it is linked to various systemic disorders [1]. One of these disorders is metabolic dysfunction-associated steatotic liver disease (MASLD), with a global prevalence of over 30% [2]. Previously known as non-alcoholic fatty liver disease (NAFLD), MASLD is the most common chronic liver disease and a leading cause of liver-related morbidity and mortality. Prevalence increases with age, body mass, and physical inactivity [3]. Male sex is also a significant risk factor [2].

MASLD imposes a significant burden on both the healthcare system and the lives of individuals. Given the association of MASLD with other metabolic conditions, primarily type 2 diabetes and obesity, it poses a significant risk for extrahepatic comorbidities, especially cardiovascular and renal diseases [4-6]. In the United States, MASLD and its more severe form, metabolic dysfunction-associated steatohepatitis (MASH), have become one of the leading causes of liver transplantation in adults. The burden of MASLD and its associated complications, such as hepatocellular carcinoma, is expected to rise in the coming years [7].

Current treatment for MASLD focuses on lifestyle modifications, including weight loss and physical activity [8]. Despite significant progress in understanding MASLD pathogenesis and advances in clinical research, there remains a paucity of approved pharmacological treatments. The first medication, resmetirom, was approved by the US Food and Drug Administration in March 2024. However, it is used only for patients with more severe forms of MASLD (noncirrhotic MASH) and is not yet widely available in many countries. Additionally, cost presents another challenge [9]. Therefore, the cornerstone of successful MASLD management is lifestyle modification and weight loss.

This review summarizes the current understanding of lifestyle modification in MASLD and pays special attention to educational approaches.

## Review

Lifestyle modification

Weight loss remains the cornerstone of MASLD management. According to the latest recommendations of the European Association for the Study of the Liver (EASL), published in 2024, a weight loss of more than 5% is recommended to reduce total liver fat, 7-10% to decrease liver inflammation, and 10% to improve fibrosis. For adults with MASLD without overweight or obesity, losing weight up to 3-5% is also recommended [8]. These indicators should be achieved by improving diet quality, increasing physical activity, and other lifestyle habits [8].

Achieving sufficient weight loss and maintaining it over the long term is a major challenge. Below, we briefly review the most evidence-based lifestyle modification methods recommended for patients with MASLD.

Diet

Balanced diets rich in whole grains, vegetables, fruits, and fish are recommended for weight loss in patients with hepatic steatosis [10]. To achieve targeted weight loss results, it is recommended to create a calorie deficit by reducing daily energy intake by 500 kcal or maintaining a total calorie intake of 1200-1500 calories per day. The most favorable nutrition pattern for patients with hepatic steatosis, according to numerous scientific studies, is the Mediterranean diet [6,8,10,11]. This diet involves eating a lot of plant-based foods with minimal processing, including olive oil, legumes, nuts, and seafood. This diet is the most studied in the context of reducing the risks of progression of hepatic steatosis as well as other metabolic diseases that accompany MASLD [12,13].

Patients with MASLD should reduce their intake of saturated fats to less than 10% of total energy, specifically from red and processed meat. Studies have shown that high consumption of saturated fat increases intrahepatic triglyceride content and has a deteriorating effect on MASLD and cardiovascular diseases [14].

A high consumption of simple sugars carries additional risks. The consumption of added sugars, particularly fructose, plays a significant role in the development of MASLD by de novo lipogenesis of saturated fats in the liver [15]. The evidence demonstrates that consuming ≥4 sugar-sweetened beverages weekly increases MASLD risk by 45% [16], with frequent intake being particularly dangerous in the context of the development of hepatic steatosis and transition from MASLD to MASH [17]. On the other hand, reduced sugar consumption improves liver fat content within a few weeks [18].

Coffee consumption, with or without caffeine, has been demonstrated to play a protective role in MASLD. A meta-analysis of 11 epidemiological studies revealed that regular coffee consumption significantly lowers the risk of MASLD compared to those who do not drink coffee [19]. Current EASL guidelines on MASLD management mention the benefits of coffee consumption [8].

Patients with MASLD who also have sarcopenia require specific dietary recommendations, including higher protein intake (1.2-1.5 g/kg) and reduced fasting between dinner and breakfast in cases of advanced liver fibrosis [12].

Physical Activity

A sedentary lifestyle is an independent predictor of MASLD and is associated with a greater risk of its progression [20]. Patients with MASLD are advised to engage in more than 150 minutes per week of moderate-intensity or 75 minutes per week of vigorous-intensity physical activity [8]. According to WHO Guidelines on Physical Activity and Sedentary Behavior, physical activity is defined as any bodily movement produced by skeletal muscles that requires energy expenditure. So, in general, “every move counts towards better health” in MASLD [21].

It has been proven that regular physical activity decreases hepatic fat accumulation and enhances peripheral insulin sensitivity. This leads to reduced adipocyte lipolysis, a lower influx of free fatty acids to the liver, and a decrease in de novo lipogenesis [22]. Various exercise training programs, including aerobic training, resistance training, high-intensity interval training, or combined approaches, with differing session frequencies, durations, and intensities, have proven effective in reducing steatosis [23]. It was shown that increased physical activity is beneficial for hepatic and cardiometabolic health, even independent of weight loss [20]. A 12-week intervention with different exercise modalities resulted in a reduction in liver fat content ranging from 13.7% to 14.3%, depending on the type of physical activity, even without significant alterations in body weight or visceral adiposity [24]. Therefore, most clinical recommendations suggest a combination of aerobic and resistance training based on individual preferences and local socioeconomic context [8].

Alcohol and Smoking

Limiting alcohol consumption and smoking is an important component in the prevention and treatment of MASLD. Moreover, both behavioral habits potentiate the numerous metabolic comorbidities that may be present in patients with MASLD. There is evidence from meta-analyses that smoking is related to MASLD and liver fibrosis and can increase the risk of liver cancer [25,26].

The role of alcohol consumption in MASLD remains controversial, with some studies suggesting that low to moderate alcohol intake is associated with reduced odds of developing MASH [27]. However, later studies, particularly longitudinal studies, have changed the view of the role of alcohol in MASLD. In one study, after a mean follow-up of 11.1 years, patients with MASLD and even low alcohol intake (10-19 g of daily general alcohol use) were associated with increased risks for advanced liver disease and cancer [28]. Therefore, alcohol consumption and smoking are highly discouraged in the population with MASLD, as clearly stated in current clinical guidelines [8].

Barriers to achieving lifestyle modification in MASLD

Barriers to a healthy lifestyle in various metabolic diseases, including MASLD, cover several diverse areas that extend beyond clinical medicine and encompass cultural factors, public health policy, healthcare systems, and living environment. There is no simple solution to overcoming MASLD. Instead, a comprehensive and holistic approach based on the patient’s individual preferences, while also considering the local socioeconomic context, can help achieve substantial results in lifestyle modification. In this review section, we analyze the role of various factors that influence the risk and progression of MASLD, such as motivation, patient education, and social nutrition principles.

Socioeconomic Factors

Many socioeconomic factors influence the development of metabolic diseases, particularly MASLD. They often intertwine and reinforce each other, so addressing these problems must be comprehensive. Low income has been linked to MASLD, although it serves as a cumulative marker of food insecurity and living in an environment with barriers to physical activity. Food insecurity refers to the persistent lack of adequate food necessary for a healthy lifestyle due to financial constraints [29]. Low-income populations tend to live in food deserts, places with significant challenges in accessing nutritious, affordable, and diverse food. These areas, characterized by limited availability of grocery stores and fresh produce, often force residents to rely on processed, high-calorie, and nutrient-poor foods from convenience stores or fast-food outlets. As a result, individuals in these communities are at a higher risk of developing diet-related health issues, including obesity, diabetes, and MASLD [10].

Another important socioeconomic factor in the development of MASLD is the living environment. Enhancing the built environment can play a crucial role in promoting a healthy lifestyle by encouraging physical activity. Research indicates that individuals living in walkable neighborhoods, featuring safe spaces for walking, cycling, and outdoor recreation, are more likely to engage in regular physical activity and have a lower risk of obesity, diabetes, and hypertension [30].

Patient Awareness and Stigma

Despite its widespread prevalence, patients and healthcare providers are largely unaware of MASLD. Many patients with hepatic steatosis remain undiagnosed, mainly because the disease has no specific symptoms, and knowledge of MASLD remains poor among non-hepatology and gastroenterology specialists [31]. Patients may perceive their condition as not serious, especially in the context of the lack of available pharmacological treatment and clear instructions from doctors. This may contribute to the fact that patients without symptoms or with mild symptoms do not receive a timely diagnosis of MASLD, which leads to the progression of metabolic problems and the development of long-term complications [32].

Another problem may be the stigma associated with a diagnosis. The previous nomenclature of the diagnosis “nonalcoholic fatty liver disease (NAFLD)” was changed to “metabolic dysfunction-associated steatotic liver disease (MASLD)” to avoid potential stigmatization. Patients may also experience judgment and misunderstanding from both society and doctors; the former often relates to suspicion of alcohol use, while the latter stems from the perception that the diagnosis is not life-threatening, since the person may appear to be in good health [32]. The terms "nonalcoholic" and "fatty" were perceived as stigmatizing by 61% and 66% of participants, respectively, in a multisociety Delphi process on new fatty liver disease nomenclature. The adoption of new, non-stigmatizing terminology is expected to enhance awareness and patient identification by enabling patients to better comprehend their condition and assisting healthcare professionals in explaining and understanding the disease more effectively [33].

Motivation

Motivating long-term lifestyle changes for obesity-related diseases such as MASLD remains one of the greatest challenges of the 21st century. Despite the awareness that weight loss is the main treatment for MASLD, most patients have relatively low motivation to make significant lifestyle changes, which may be influenced by many factors [34]. Key barriers include insufficient knowledge about the nature of steatotic liver disease, its predominant asymptomatic course, chronic stress, and lack of social support. Conversely, social support, the emergence of symptoms, and concerns about long-term health consequences can drive behavior changes aimed at weight loss [34,35]. Accordingly, lifestyle interventions for MASLD should be individualized, accessible, and culturally appropriate. To increase motivation, patients should be educated about the nature of the disease and involved in developing nutritional, physical, behavioral, and other components of the treatment plan. Long-term care involves multidisciplinary teams as well as social support from family, friends, and caregivers [32].

Approaches to lifestyle modification in MASLD

Education regarding lifestyle modification in patients with MASLD can be delivered in a variety of ways. Some are more traditional, such as an individual visit to a healthcare provider, and some involve more modern approaches, such as web-based programs or the use of smartphone applications. Regardless of the method, it is important to achieve weight loss goals by improving the quality of the diet and increasing physical activity. Various methods and their combinations may be useful here.

Individual Health Visit

Traditional medical visits play a crucial role in the management of MASLD, especially in primary care. Regular consultations with healthcare providers facilitate early detection in high-risk populations, monitoring of disease progression, and implementation of lifestyle interventions, such as weight loss, dietary modifications, and physical activity, which are cornerstone strategies for managing MASLD [36]. These visits also allow for the management of comorbid conditions like obesity, type 2 diabetes, and dyslipidemia, which are often overlapping and closely linked to MASLD progression [37]. Long-term adherence to medical advice and lifestyle changes can reduce liver fat, improve fibrosis, and lower the risk of adverse outcomes such as cirrhosis, hepatocellular carcinoma, and cardiovascular disease [38]. However, patient adherence to lifestyle modifications, such as dietary changes and physical activity, is often suboptimal due to a variety of socioeconomic factors, lack of motivation, and insufficient follow-up support from healthcare providers. Additionally, challenges in access to healthcare can limit the effectiveness of these interventions, highlighting the need for integrated, multidisciplinary approaches to optimize long-term outcomes [10].

Several behavioral interventions can significantly enhance weight loss and help patients with MASLD achieve their lifestyle goals. Techniques such as self-monitoring, goal setting, and stimulus control have been shown to improve adherence to dietary and physical activity recommendations. Self-monitoring, including food diaries and activity logs, helps individuals track progress and identify patterns that may hinder weight loss [39,40]. Additionally, cognitive-behavioral strategies, such as problem-solving and relapse prevention, can address emotional and environmental triggers that lead to overeating or sedentary behavior. Combining these tools with personalized feedback from healthcare providers can further enhance long-term adherence and success in weight management [41].

Group-Based Approach in MASLD

Another effective strategy for MASLD, offering structured support and fostering accountability among participants, is group-based programs. These programs often combine education, behavioral interventions, and peer support to promote lifestyle changes such as weight loss, improved diet, and increased physical activity. A study by Promrat et al. (2010) demonstrated that a 48-week group-based lifestyle intervention significantly reduced liver fat and improved liver histology in patients with MASH [42]. Social support and shared experiences within group settings also help mitigate feelings of isolation and improve long-term compliance with lifestyle modifications [43]. These findings highlight the potential of group-based programs as a scalable and cost-effective approach to managing MASLD. Furthermore, group-based programs can be delivered virtually through telehealth, and there is evidence that even short-term interventions, such as a six-session program, can be beneficial in the management of MASLD [44].

Web-Based Intervention in MASLD

The shift toward digital healthcare presents a promising opportunity for MASLD management, offering accessible, scalable, and personalized interventions. This gives substantial promise in managing MASLD by providing accessible, scalable, and personalized digital interventions. A systematic review and meta-analysis published in 2023 evaluated the effectiveness of digital health technologies, including internet-based programs, telephones, and mobile apps, in promoting lifestyle modifications for patients with MASLD. The findings demonstrated that eHealth interventions significantly improved body mass index (BMI), alanine aminotransferase (ALT), and aspartate aminotransferase (AST) levels, highlighting their potential as scalable tools for MASLD management [45].

One study compared group-based (n=438) and internet-based (n=278) approaches to lifestyle changes in patients with MASLD. After two years of follow-up, internet-based counseling was found to promote significant weight loss in motivated patients and was as effective as group counseling [46]. It was also shown that web-based intervention is as effective as a group-based intervention in reducing the long-term risk of diabetes development in individuals with MASLD, primarily through comparable weight loss outcomes. Both interventions led to similar reductions in BMI and diabetes incidence [47].

Another study of an eight-week individualized web-based exercise program demonstrated a significant reduction in non-invasive markers of hepatic steatosis, inflammation, and fibrosis in patients with MASLD. Improvements included reductions in liver enzymes (ALT and AST), inflammatory markers (hsCRP and ferritin), and fibrosis scores (FLI, FIB-4, and APRI), alongside enhanced health-related quality of life, highlighting the potential of digital exercise interventions for MASLD management [48].

Mobile Technologies in MASLD

Several studies have demonstrated the important role of using mobile technologies in modifying the lifestyle of patients with MASLD. In one randomized controlled trial, it was shown that using a special mobile application in the intervention group demonstrated a significantly higher likelihood of achieving ≥5% weight loss at six months, with a relative risk of 5.2 (P=0.003, 95% CI 1.8-15.4), representing a fivefold increase compared to the control group. The intervention also led to improvements in anthropometric indices, such as waist circumference and body weight, along with reductions in liver enzyme levels [49].

Another study from Thailand demonstrated that providing healthy lifestyle information and MASLD education through a social media application (LINE) significantly improved liver stiffness in patients with MASLD compared to standard of care, highlighting the potential of social media platforms to enhance clinical outcomes in MASLD management [50].

Limitations in Lifestyle Modification in MASLD

**Figure 1:**
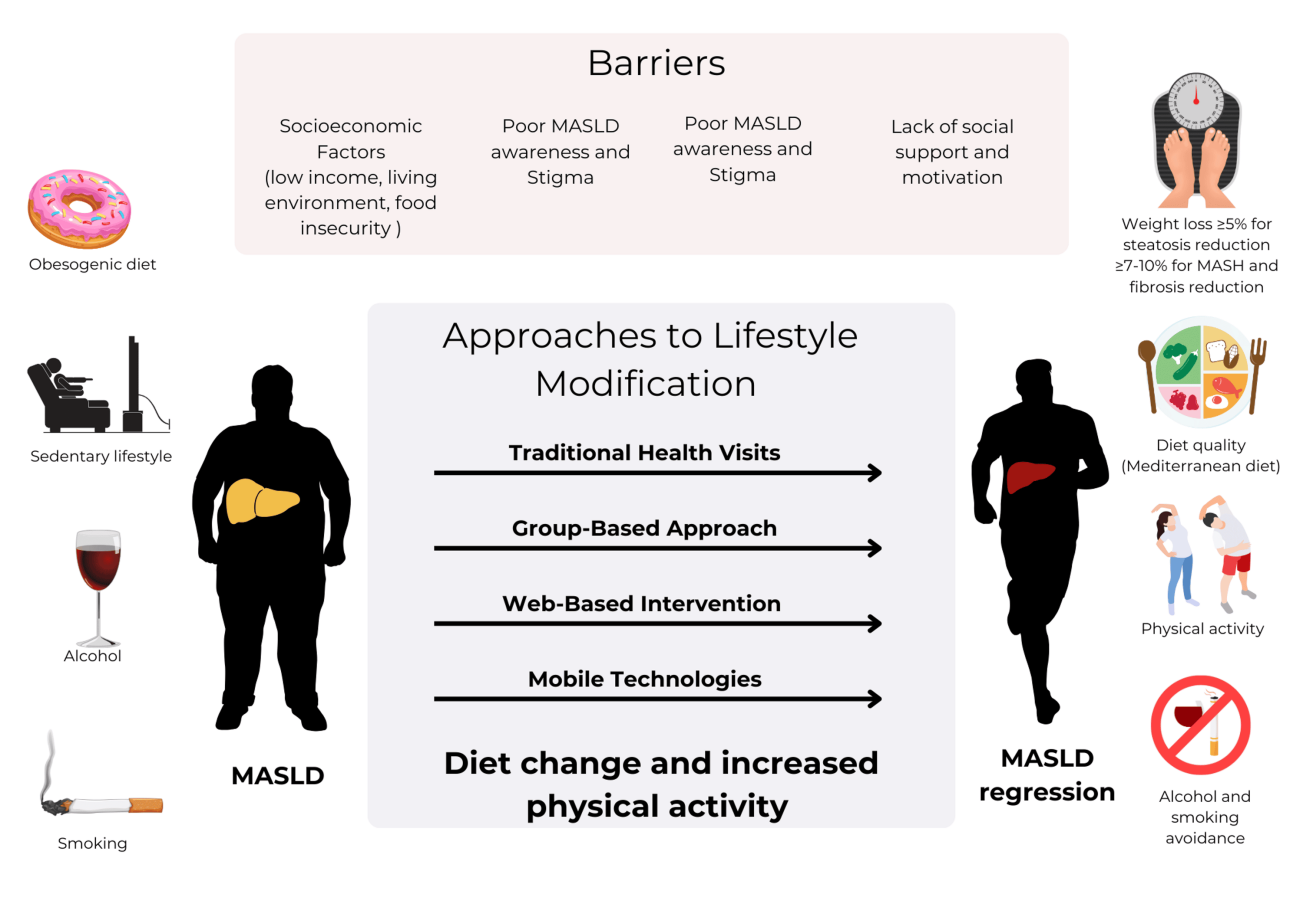
Conceptual model of risk factors, barriers, and strategies for lifestyle modification in MASLD MASLD, metabolic dysfunction-associated steatotic liver disease; MASH, metabolic dysfunction-associated steatohepatitis Image credit: Created by the author (Stanislav Kravchuk).

Lifestyle modification remains the cornerstone of MASLD management, but its implementation faces several limitations, including patient adherence, healthcare provider communication skills, accessibility, and individual variability in response to interventions. Web-based approaches, while promising, are not universally applicable, as they tend to be more effective for men, younger individuals with higher education levels, and those comfortable with technology [46]. Additionally, studies on web-based exercise programs, though showing positive outcomes such as improved liver enzyme levels and reductions in steatosis and fibrosis, have been limited by small sample sizes and single-arm designs, highlighting the need for larger, more robust trials to confirm their efficacy and scalability. These limitations underscore the importance of personalized, accessible, and inclusive strategies to optimize lifestyle interventions for diverse MASLD populations [10]. A conceptual model summarizing the key risk factors, barriers, and strategies for lifestyle modification in MASLD is presented in Figure 1.

## Conclusions

MASLD is a growing global health challenge, closely linked to obesity, type 2 diabetes, and sedentary lifestyles. As the most common chronic liver disease, it significantly contributes to both liver-related and extrahepatic complications, including cardiovascular disease and chronic kidney disease. While pharmacological treatments like resmetirom offer hope for severe cases, lifestyle modification remains the cornerstone of MASLD management for billions of patients worldwide. Lifestyle interventions should be based mainly on a healthy diet and physical activity, aiming for weight loss of more than 5% to reduce total liver fat, and 7-10% to decrease liver inflammation and promote fibrosis regression. This review emphasizes evidence-based lifestyle interventions, such as adopting a Mediterranean diet, reducing saturated fats and added sugars, and increasing physical activity. It also highlights the need to address socioeconomic barriers, patient awareness, and stigma, which often hinder effective lifestyle changes. Innovative approaches, including group-based programs, web-based interventions, and mobile technologies, show promise in delivering scalable and personalized care.

A multidisciplinary, holistic approach is crucial for optimizing MASLD management. This includes patient education, behavioral strategies, and social support to enhance motivation and adherence. Healthcare providers must be equipped to communicate the importance of lifestyle changes and address diverse patient needs. As MASLD prevalence rises, prioritizing lifestyle interventions and addressing systemic barriers will be key to reducing its global impact and improving outcomes. This is particularly important at the primary care level and should be supported by health policies. Future research should focus on refining these strategies and ensuring their accessibility for all affected individuals.
